# SCL-90-R Symptom Profiles and Outcome of Short-Term Psychodynamic Group Therapy

**DOI:** 10.1155/2013/540134

**Published:** 2013-04-27

**Authors:** Hans Henrik Jensen, Erik L. Mortensen, Martin Lotz

**Affiliations:** ^1^Medical Psychology Unit, Department of Environmental Health, Faculty of Health and Medical Sciences, Institute of Public Health, University of Copenhagen, Øster Farimagsgade 5A, P.O. Box 2099, 1014 Copenhagen, Denmark; ^2^Psychiatric Department E, Bispebjerg Hospital, Copenhagen, Denmark

## Abstract

*Background*. Psychodynamic group psychotherapy may not be an optimal treatment for anxiety and agoraphobic symptoms. We explore remission of SCL-90-R Global Severity Index (GSI) and target symptoms in 39 sessions of psychodynamic group therapy. *Methods*. SCL-90-R “target symptom” profile and GSI remission according to Danish norms were identified in 239 patients and evaluated according to reliable and clinical significant change. *Results*. Four major groups of target symptom cases (depression, interpersonal sensitivity, anxiety, and phobic anxiety) covered 95.7% of the sample. As opposite to phobic anxiety and anxiety patients, patients with interpersonal sensitivity obtained overall the most optimal outcome. The phobic anxiety scale, social network support, and years of school education were independent predictors of GSI remission, and a low anxiety score and absence of phobic anxiety target symptoms were independent predictors of remission of target symptom pathology. *Conclusions*. The negative results as associated with the SCL-90-R phobic anxiety scale and the phobic anxiety target symptom group are largely in agreement with recent studies. In contrast, whatever the diagnoses, patients with interpersonal sensitivity target symptom may be especially suited for psychodynamic group therapy. The SCL-90-R subscales may allow for a more complex symptom-related differentiation of patients compared with both diagnoses and GSI symptom load.

## 1. Introduction

 A review of analytic and psychodynamic group therapy provides evidence for the effectiveness of treatment in groups, but not necessarily for the superiority of analytic and psychodynamic group therapy [1]. In a recent randomized controlled trial, Watzke et al. selected patients for treatment in either psychodynamic or cognitive behavioral groups [2]. Selection for psychodynamic treatment included evaluations of psychological mindedness and the ability to work with nonsymptomatic treatment goals (e.g., “the integration of unconscious experience” and to “allow experience of emotions”), whereas treatment goals that focused on coping with specific situations and ICD-10 anxiety diagnoses qualified for cognitive behavioral therapy (ibid, page 97). Outcome was evaluated with the Symptom Check List-14 Global Severity Index (GSI) which is a short form of the SCL-90-R [3]. Watzke et al. concluded that consecutive unselected admission of patients favored a cognitive behavioral approach, whereas psychodynamic therapy requires specific selection if comparable improvement in global symptom distress should be obtained. This result made Fonagy recommend a closer attention to the needs of the individual patients in psychodynamic therapy, among them symptomatic improvement [4]. 

 Recently, Kirchmann et al. analyzed pretreatment GSI-corrected SCL-90-R subscale profiles in a heterogeneous sample of patients in psychodynamic group therapy [5]. The study identified several pretreatment clusters, including “insecure phobic” patients with elevated “interpersonal sensitivity” and “phobic anxiety” symptoms. These patients had a significantly less favorable outcome of the treatment as evaluated with the GSI, the SCL-90-R depression subscale, and the Inventory of Interpersonal Problems (IIP). Outcome measures were expressed as residual gain scores that correct for differences in pretreatment levels. However, differences between symptom profile clusters were small and “rather of theoretical importance than clinical relevance” (ibid, page 80).

 The aim of the present study is to explore the utility of SCL-90-R pretreatment subscale scores in outcome evaluations of 39 sessions of psychodynamic group psychotherapy. The SCL-90-R is a multidimensional questionnaire developed to screen for a range of psychological symptoms and psychopathological features on nine subscales: somatization, obsessive compulsion, interpersonal sensitivity, depression, anxiety, hostility, phobic anxiety, paranoid ideation, and psychoticism. However, the validity of using the SCL-90-R as a screening instrument on the subscale level in both psychiatry and somatic medicine has been controversial, and both exploratory and confirmatory factor analyses have failed to confirm the original nine-factor structure [6]. Consequently, in psychotherapy outcome research, the SCL-90-R has been best conceptualised as a one-dimensional measure of symptom load and the Global Severity Index (GSI)—the summary measure of the nine subscales—has become the overall most widely used measurement of psychological distress [7].

 However, in agreement with Kirchmann et al. [5], there is indication that the items of the test contain substantially more information than the GSI utilizes [8]. In contrast to GSI-corrected (i.e., ipsatized) subscale scores as described by Kirchmann et al. [5], we classify patients according to the most salient SCL-90-R subscale score (the “target symptom”). Furthermore, the most salient subscale score should be above the Danish norm for caseness [9]. “Target symptoms” usually refer to symptoms of an illness that are most likely to respond to a specific treatment (i.e., antidepressants for depression symptoms) [10]. However, target symptoms have also been conceptualized as the most distressing problems, or primary complaints, presented in the clinical interview before therapy, which have probably motivated the seeking treatment [11, 12]. Consequently, from the patient perspective, target symptom improvement may be some of the most obvious evaluations of outcome in psychotherapy research.

Geiser et al. found outcome of psychodynamic group therapy to be less favorable for anxiety patients as measured with the SCL-90 phobic anxiety scale, unless treatment was supplemented with behavioral exposure of agoraphobic symptoms [13]. Consequently, in the present study—and in agreement with Watzke et al. [2] and Kirchmann et al. [5]—we expect patients with anxiety symptoms as measured with the SCL-90-R subscales to be less likely to improve. Improvement in target symptoms and GSI is evaluated according to Jacobson and Truax's constructs of reliable and clinical significant change (CSC) [14]. “Remission” is obtained when a patient reliably improves below the Danish cutoffs for SCL-90-R dysfunction. To our knowledge, there have been no previous psychotherapy evaluations based on classification and outcome of pretreatment SCL-90-R target symptoms groups. 

## 2. Methods

### 2.1. Participants

The study is part of a long-term outpatient naturalistic psychotherapy evaluation of the outpatient psychotherapy unit, Bispebjerg Hospital, Copenhagen, Denmark. Through a five-year period 378 patients received 39 sessions of psychodynamic group therapy. In agreement with the psychodynamic approach, the groups were composed of heterogeneous patient samples [15]. Of these patients, 348 (92%) accepted to participate in the evaluation project. However, four had missing pretreatment SCL-90-R data, and 15 had subscale scores that did not fulfil the Danish SCL-90-R norm criteria for caseness [9]. Of the 329 patients included in the present analyses, 90 patients (27.4%) did not respond to posttreatment questionnaires. Consequently, 239 patients were available for outcome analyses yielding a participation rate of 63.2% (239/378).

 At referral, the patients most often had a tentative diagnosis. They were invited to a first interview (including systematic registration of demographic and psychiatric variables) with one of the psychiatrists or clinical psychologists. This interview was discussed at a conference confirming or revising the initial diagnosis. The diagnosis was further established while the patient was participating in a four-day group-based introduction to the department and to group psychotherapy, and concordance between first and final diagnosis was 85%. Patients with dementia, psychosis, and drug or severe alcohol abuse (that disqualified for the present treatment) were referred to other departments. 

### 2.2. Therapists and Treatment

Two therapists and 6–8 patients participated in each of the four slow-open heterogeneous groups. The majority of the therapies were administered by nonacademic staff (nurses, social workers, and occupational and physical therapists) with a psychiatrist, a physician (under training), or a psychologist as cotherapist. All therapists had some or full psychotherapeutic training (gestalt, family, cognitive behavioral, or analytic group psychotherapy), and all therapies were conducted under supervision within a psychodynamic group reference. About half of the staff had specific training or formal education in group psychotherapy, and about one-fourth had specific training in psychodynamic group therapy.

 For each patient, a psychodynamic focus was established in the introduction group after a thorough examination. The individual final focus formulation was suggested by the leader, an experienced psychoanalyst (M. Lotz). If an unconscious component could not be easily defined, a more superficial, rational, and practical focus was used. The focus did not include any direct symptomatic improvement. The recommendation was that the therapists should intervene to keep the members of the group working with the difficulties of each member. Confronting and interpreting the resistance—for example passivity or silence—was the most important job for the therapists. To be in contact with emotionality was weighted higher than just neutral memories, and the here-and-now was weighted higher than a psychogenetic understanding. There was no manual for the therapists. The attitude was to have the psychodynamic focus in mind, while letting the associations flow quite freely.

### 2.3. Measurement

Psychiatric and sociodemographic variables were collected at a diagnostic pretreatment interview. The Symptom Check List-90-R (SCL-90-R) was answered within two weeks before and after treatment. Derogatis recommended that a patient should be considered a “case” when scoring higher than the norm population on the Global Severity Index (GSI), or, alternatively, when the subscale cutoff is exceeded in scores on two (or more) subscales [3]. Target symptom caseness was defined according to Derogatis criteria (ibid), that is, a score at or above a T-score of 63 according to Danish gender specific norms [9]. 

### 2.4. Statistical Analysis

Pre/posttreatment improvement was measured as difference scores (posttreatment minus pre-treatment), and as residual gain scores that correct for the pretreatment level of pathology [16]. 

 According to Jacobson and Truax, the reliable Change Index (RCI) is viewed as the minimal individual pre/posttreatment change (difference score) that may be called “statistically significant” [14]. Cutoffs for SCL-90-R reliable changes may be calculated from Cronbach's alpha which, as evaluated in a Danish norm population [9], revealed coefficients ranging from .73 (hostility and psychoticism) to .91 (depression) with a mean of .83. The RCI ranged from .42 (phobic anxiety) to .71 (paranoid ideation) with a mean of .54. Patients obtained clinical significant change (CSC) status when they reliably improved below the Danish cutoffs for caseness (ibid). Similarly, improvement in the Global Symptom Index (GSI) was classified according to Jacobson and Truax [14] and Danish norms [6]. 

 All statistical analyses were conducted with SPSS version 18.0 (SPSS for Windows Inc., Chicago, IL, USA). For significance tests, alpha was set at .05.

## 3. Results 

The patients were 36.7 (SD 11.1) years of age with 7.1 (9.1) years' duration of illness, and 73% were women (see Jensen et al. for further description of the population) [17–19]. All patients had ICD-8 diagnoses, and 35.4% had both ICD-8 and ICD-10 diagnoses. According to accepted guidelines for ICD-8/ICD-10 conversion [20], reclassification of patients with ICD-8 diagnoses showed that the sample included 1 patient (.4%) with substance abuse (*F*10–19), 3 (1.2%) with schizophrenia, schizotypal and delusional disorders (in remission) (*F*20–29), 22 (9.2%) with mood (affective) disorders (*F*30–39), 123 (51.5%) with neurotic, stress-related, and somatoform disorders (*F*40–48), 1 (.4%) with eating disorders (*F*50), and 89 (37.2%) with personality and behavior disorders (*F*60–69). 

### 3.1. Target Symptom Groups

Depression, anxiety, interpersonal sensitivity, and phobic anxiety target symptom patients represented 95.7% of the 239 project completers, whereas 3 (1.3%) were somatization, 3 (1.3%) hostility, 2 (.8%) obsessive compulsion, 2 (.8%) psychoticism, and none paranoid ideation target symptom cases. However, one of the somatization and one of the hostility target symptom patients only had case status on one scale. Consequently, we only included the four major target groups in the outcome analyses below (*n* = 229) covering for 53.3% (122) with depression, 19.2% (44) with anxiety, 17.5% (40) with interpersonal sensitivity, and 10.0% (23) with phobic anxiety target symptoms of the included patients. 

 The target symptom classification was not related to gender (Chi-square 2.16, *P* = .5), age, or duration of illness (one-way ANOVA, *F* < 1.84, *P* > .1). However, anxiety and phobic anxiety patients were significantly more likely to receive medication at pretreatment as compared with depression and interpersonal sensitivity patients (61.2% versus 44.0%; Chi-square 5.56, *P* < .05). 

 The mean (SD) pre- and posttreatment scores are shown in Table 1, first column. Within each target group, the mean score of the target symptom scale was at least one SD (of the GSI distribution) above the mean scores of each of the other subscales. At pretreatment the phobic anxiety group had a significant higher target symptom score compared with the other groups (one-way ANOVA, Tukey post hoc test; *F*14.1, *P* < .001), but this was not the case for GSI (one-way ANOVA, *F*2.44, *P* = .07). 

### 3.2. Outcome

Anxiety and phobic anxiety patients did not differ from depression and interpersonal sensitivity patients in medication status at posttreatment (26.6% versus 37.1%; Chi-square 2.22, *P* > .1). Moreover, the phobic anxiety and interpersonal sensitivity patients had the highest and lowest posttreatment target symptom score, respectively (one-way ANOVA, Tukey post hoc test; *F*4.69, *P* < .01), but even though the overall test for posttreatment GSI was significant (*P* = .04), the post hoc test revealed no significant mean differences.

 Improvement as indicated by target symptom difference scores was significant (one-way ANOVA, Tukey post hoc test; *F* > 2.74, *P* < .05). Due to differences in pretreatment scores, we analysed residual gain scores. In this analysis, the interpersonal sensitivity target symptom group had the largest improvement in interpersonal sensitivity and obsessive compulsive symptoms (one-way ANOVA, Tukey post hoc test; *F* > 2.84, *P* < .05). 

Table 1, right column, presents outcome within the four major target symptom groups according to Jacobson and Truax [14]. Differences between groups were significant (Fisher's exact test 36.9 *P* < .001; post hoc Fisher's exact test > 6.6, *P* < .05), except for the depression versus interpersonal sensitivity contrast (Fisher's exact test .9 *P* = .6). Interpersonal sensitivity target patients were the most likely, with phobic anxiety patients being the least likely, to obtain target symptom CSC end-state status (Chi-square > 5.15, *P* < .05).

 As can be seen from Table 1 (bottom lines), target symptom and GSI remission were achieved by 25.8% and 29.7% of the patients, respectively. GSI and target symptom severity were significantly correlated (Pearson *r* = .76), but target groups did not differ in GSI improvement (Chi-square 6.46, *P* = .37). However, 33.8% of those with GSI CSC end-state status were still target symptom cases, ranging from 100% in phobic anxiety, 33.3% in anxiety, 29.7% in depression, to 18.8% in interpersonal sensitivity patients, respectively. Overall, interpersonal sensitivity and depressed patients obtained the best results according to CSC.

### 3.3. Logistic Regression

Variables that reached significance in bivariate analyses of GSI remission were analysed in a logistic forward Wald stepwise regression procedure in subsequent blocks of sociodemographic, psychiatric, and SCL-90-R variables (*P* < .05 as inclusion and *P* > .1 for exclusion criteria). 

 GSI remission was significantly associated with longer school education (10.0 years (1.5) versus 10.6 years (1.6)), having relevant occupation (including all kinds of participation in education, social programs, and voluntary work; 23.1% versus 40.7%), having social network support (17.9% versus 35.2%), lower GSI (1.77 (.50) versus 1.53 (.35)), and lower somatization (1.05 (.61) versus .82 (.49)), anxiety (2.14 (.73) versus 1.77 (.53)), hostility (1.24 (.76) versus .99 (.80)), phobic anxiety (1.60 (1.07) versus 1.07 (.85)), and psychoticism (1.23 (.54) versus 1.05 (.50)) subscale scores. Independent predictors in the regression procedure were the phobic anxiety scale (Wald 10.54; OR .54), social network support (Wald 9.72; OR 3.16), and years of school education (Wald 4.03; OR 1.39) explaining 6.9% of variance (Nagelkerke pseudo R-square). 

 Target symptom remission was significantly associated with lower age (37.8 (11.1) versus 34.2 (10.9)), higher prevalence of women (15.0% men versus 29.6% women), longer school education (10.0 years (1.6) versus 10.7 years (1.6)), having relevant occupation (21.0% versus 33.7%), lower prevalence of ICD-8 anxiety neuroses (29.7% versus 16.9%), lower GSI (1.79 (.47) versus 1.43 (.39)), lower somatization (1.07 (.58) versus .72 (.50)), obsessive compulsive (1.33 (.47) versus 1.13 (.46)), anxiety (2.18 (.67) versus 1.61 (.60)), hostility (1.25 (.80) versus .93 (.92)), phobic anxiety (1.64 (1.07) versus .88 (.87)), paranoia (1.29 (.77) versus 1.01 (.64)), and psychoticism (1.24 (.52) versus 1.01 (.51)) subscale scores, with absence of phobic anxiety target symptoms (28.6% versus 0%), but a higher prevalence of interpersonal sensitivity target symptoms (22.8% versus 40.0%). Independent predictors were the anxiety scale (Wald 21.82; OR .42), and phobic anxiety target symptoms (OR .00) explaining 23.6% of variance. 

 Bivariate analyses of ipsatized SCL-90-R subscale scores as employed by Kirchmann et al. [5] corresponded largely with the bivariate analyses of subscales as described above. However, none reached significance in the forward regressions and, consequently, did not alter the final predictions. 

### 3.4. Diagnoses

The ICD-10 diagnostic classification of target symptom groups is shown in Table 2. Two patients had schizophrenia, schizotypal, and delusional disorders in remission (*F*20) and one had an eating disorder (*F*50). These patients are shown on the left side in the table. Analysis of the three major diagnostic categories of mood (*F*30), neurotic (*F*40), and personality disorders (*F*60) revealed significant differences (Fisher's exact test, *P* < .001). Except for the interpersonal sensitivity versus depression contrast (*P* = .36), all post hoc comparisons were significant (Fisher's exact test, *P* < .05). Phobic anxiety and anxiety target symptom patients were significantly more likely to have neurotic disorders (*F*40), and depression and interpersonal sensitivity target symptom patients and significantly more likely to have personality disorders (*F*60). It should, however, be noted that almost one-third of the anxiety target symptom patients had a personality disorder, whereas all except one of the phobic anxiety patients had neurotic disorders (73.9% corresponding to ICD-8 “anxiety neurosis”). 

 Analyses of residual gain within ICD-10 neurotic or personality disorder diagnoses revealed that interpersonal sensitivity target patients improve the most as compared with other target symptom groups (one-way ANOVA, Tukey post hoc test; *F* > 4.33, *P* < .05). Improvements were seen on the interpersonal sensitivity, obsession compulsion, anxiety, phobic anxiety, and psychoticism scales. Overall, interpersonal sensitivity target symptom patients had residual gain scores ranging from −.55 to −.22, as compared with mostly positive values in other patients (ranging from −.14 to .20). The negative algebraic signs indicate that interpersonal sensitivity patients improve more than would be expected from their baseline scores. 

### 3.5. Project Compliance

Ninety patients (27.4%) of the eligible 329 patients did not respond to posttreatment questionnaires. Associations between dropout of the project and ICD-10 diagnosis, SCL-90-R GSI, subscale scores, ipsatized subscale scores, and target symptom groups were tested in bivariate analysis. ICD-10 personality disorders (37.6% (92) versus 50.0% (42)), depression target symptoms (51.0% (128) versus 35.7% (30)), phobic anxiety target symptoms (9.8% (24) versus 20.2% (17)), paranoid ideation target symptoms (.0% (0) versus 6.0% (5)), hostility target symptoms (1.6% (4) versus 6.0% (5)), and ipsatized interpersonal sensitivity scores (mean .04 (SD .52) versus .18 (.57)) were significantly associated with dropout (Chi-square and Fisher's exact test, *P* < .05; independent samples *t*-test, *t* = 2.01, *P* = .04). Thus, the final group of compliant patients as analyzed in the study was biased due to a selective dropout of the project. 

## 4. Discussion

In the present study of 39 sessions of psychodynamic group therapy, almost 30 percent of the patients improved into the Danish functional range of global symptom distress as evaluated with the SCL-90-R Global Severity Index, and 26% improved in target symptom pathology. Anxiety and phobic anxiety target symptom patients—representing 28% of the sample—had the worst outcome, and only 10% remitted with respect to target symptoms, as compared with 32% of depression and interpersonal sensitivity patients. Target symptom groups were not related to either GSI remission or to GSI difference or residual gain scores. However, a low pretreatment score on the phobic anxiety scale as well as a supporting network and longer school education, were independent predictors of global symptom remission, and a low anxiety score and absence of phobic anxiety target symptoms were independent predictors of remission of target symptom pathology. Moreover, phobic anxiety target symptom patients were significantly more likely to drop out of the research project. This is in contrast to the depression target group and ipsatized interpersonal sensitivity scores which were significantly related to compliance. Studies have shown that dropout of psychotherapy research projects is significantly associated with a less favorable outcome of treatment [21].

 Our results are partly in agreement with those of Watzke et al.'s who described favorable outcome of psychodynamic groups as evaluated with the GSI, but only when patients with ICD-10 anxiety diagnoses and specific treatment goals were not included in psychodynamic treatment, but in cognitive behavioral therapy [2]. Similarly, our results are partly in agreement with those of Kirchmann et al.'s who found that “overly avoidant” patients with an elevated GSI-corrected phobic anxiety and interpersonal sensitivity cluster profile obtain the overall least positive outcome in psychodynamic groups [5]. 

 In the present study, however, the interpersonal sensitivity target symptom patients had the overall lowest numeric phobic anxiety score (1.16) as compared with the remaining sample (1.50). These patients obtained the overall most positive outcome as indicated by the proportion of patients with clinically significant target symptom and GSI improvement (40%), with improvement in target and obsessive-compulsive symptoms, even after adjustment for baseline levels. Moreover, interpersonal sensitivity patients, whether having neurotic or personality disorder diagnoses, had the overall largest improvement on several of the SCL-90-R subscales, including anxiety and phobic anxiety, as compared with other target symptom patients with similar diagnoses. Thus, whatever the pathology according to diagnosis, patients with interpersonal sensitivity target symptoms, but without substantial agoraphobic avoidance as indicated by the SCL-90-R phobic anxiety scale [3], seem to be adequately treated in a group psychotherapeutic setting. This is in agreement with Lorentzen and Høglend who found the interpersonal sensitivity scale to be positively associated with patients' and therapists' retrospective evaluations of global improvement after long-term group analytic therapy [22].

 The interpersonal sensitivity scale measures feelings of discomfort, inadequacy, and inferiority during social interaction [3]. Thus, awareness of at least some degree of interpersonal tension may be necessary for optimal outcome of psychodynamic group therapy which primarily focuses on interactions between group members. Similarly, Lorentzen and Høglend suggested that the interpersonal sensitivity scale may reflect trait-like dimensions that are amenable to change in a benign group atmosphere [21]. This may also be in agreement with Gibbons et al. who found self-understanding of interpersonal patterns to be associated with progress in psychodynamic psychotherapies [23].

 In contrast to interpersonal sensitivity, severe agoraphobic avoidance and anxiety seem not to be sufficiently addressed in the present group therapy format. This is in agreement with Geiser et al. who reported a mean pretreatment SCL-90 phobic anxiety score of 2.5 in a German sample of panic and agoraphobic patients in psychodynamic group therapy [13]. However, after combining group treatment with symptom oriented exposure, the effect size increased from .51 to 1.34, and the phobic anxiety score decreased to .80 at discharge (ibid, Figure 2, page 63). In comparison, none of the phobic anxiety patients in the present study remitted in agoraphobic avoidance, and even though 87% of the phobic anxiety patients reliably improved (Table 1), the mean end-state score was 2.05 which is 5.65 SD above the Danish normal mean [9]. 

 It is to be expected that dysphoric mood, lack of energy, lack of motivation, and demoralization are common symptoms in patients seeking treatment for psychological problems [24]. More surprisingly, however, was the fact that the depression target symptom group included almost half of the total sample of patients, whereas mood disorder diagnoses only represented 9.2%. More than eighty percent of the depression target symptom patients had neurotic or personality disorders, which may suggest a considerable amount of patients with comorbid depression. However, only 13% of the sample had double diagnoses, and none had a diagnosis of comorbid depression. These issues will be further explored in subsequent analyses, but the present finding nevertheless points to the need of formal diagnostic procedures and tests for interrater reliability, which is one of the major drawbacks in the present study. However, diagnoses were carefully considered and reevaluated during the course of therapy, which probably reflects clinical practice in most public treatment settings [2]. 

 ICD-10 diagnoses—which were available for 35.4% of the patients—may represent a more comprehensive assessment than ICD-8, especially for anxiety and personality disorder classification. Studies have shown that the phobic anxiety subscale has demonstrated a better diagnostic efficacy as compared with other SCL-90 subscales [25]. However, the total sample of phobic anxiety target symptom patients with ICD-10 diagnoses in the present study included agoraphobia, panic disorder, generalized anxiety disorder, adjustment disorder, and emotional unstable and unspecified personality disorder diagnoses. Similarly, interpersonal sensitivity target symptom patients had ICD-10 diagnoses of several specific disorders of personality, and also depression, dysthymia, generalized anxiety, mixed anxiety and depression, obsessive-compulsion, hypochondria, and bulimia. The heterogeneity of diagnoses within target symptom groups may partly explain why diagnostic classification has generally been found to be unrelated to outcome in psychodynamic psychotherapy [26, 27].

## 5. Conclusion 

Due to our clinical impressions, preliminary analyses of the present project and the available literature, we have now established a cognitive behavioural treatment program for severe anxiety patients. In this context, and in agreement with Kirchmann et al. [5], analyses of pretreatment SCL-90-R subscale data may allow for a more comprehensive symptom-related differentiation of patients as a supplement to diagnoses and other data in treatment planning. However, even though the SCL-90-R Global Severity Index is the most commonly used outcome measure in psychotherapy research [7], few studies include information from SCL symptom scales. This, of course, assumes satisfactory reliability of the SCL-90-R subscales, but Derogatis reported high internal consistency (Cronbac's alpha) ranging from .77 for psychoticism to .90 for depression in an American sample [3], and Olsen et al. reported that all SCL symptom scales—except psychoticism—had acceptable psychometric characteristics as evaluated by item response models in a Danish population [28]. Consequently, from a psychometric point of view, if the Global Severity Index is measured, it is obvious to take advantage of the SCL subscale information as well. 

## Figures and Tables

**Table 1:** Present pre- and posttreatment means (SD), and Jacobson and Truax change status groups of target symptoms and GSI for the four major target groups. Groups are arranged according to percent of patients who remitted in target pathology.

	Mean (SD) scores		Jacobson and Truax outcome groups			Total *n*
	Pretreatment	Posttreatment	No change	Reliable change	Clinical significant change	
SCL-90-R target symptoms						
Interpersonal sensitivity	2.44 (.53)	1.51 (.74)	37.5% (15)	22.5% (9)	40.0% (16)	40
Depression	2.67 (.53)	1.98 (.85)	43.5% (53)	27.0% (33)	29.5% (36)	122
Anxiety	2.38 (.54)	1.64 (.62)	36.4% (16)	47.7% (21)	15.9% (7)	44
Phobic anxiety	3.22 (.58)*	2.05 (.99)	13.0% (3)	87.0% (20)	0.0% (0)	23
SCL-90-R global severity (GSI)						
Interpersonal sensitivity	1.56 (.43)	1.04 (.50)	22.5% (9)	37.5% (15)	40.0% (16)	40
Depression	1.75 (.47)	1.34 (.64)	35.2% (43)	34.4% (42)	30.3% (37)	122
Anxiety	1.64 (.51)	1.29 (.59)	40.9% (18)	38.6% (17)	20.5% (9)	44
Phobic anxiety	1.82 (.47)	1.37 (.61)	26.1% (6)	47.8% (11)	26.1% (6)	23
Total sample						
Target symptom improvement	2.63 (.58)	1.84 (.83)	38.0% (87)	36.2% (83)	25.8% (59)	229
Global severity index (GSI)	1.70 (.47)	1.28 (.61)	33.2% (76)	37.1% (85)	29.7% (68)	229

*pretreatment score is significantly different from the remaining sample (one-way ANOVA, Tukey post hoc test).

**Table 2:** ICD-10 major diagnostic categories. Percent (*n*) within SCL-90-R target symptom groups. The distribution of diagnoses was significantly different across target symptom groups (see text) (Fisher's exact test, *P* < .001).

	ICD-10 diagnoses					
	*F*20	*F*50	*F*30	*F*40	*F*60	Total
SCL-90-R target symptoms						
Interpersonal sensitivity	5.0% (2)	2.5% (1)	7.5% (3)	35.0% (14)	50.0% (20)	100% (40)
Depression	.8% (1)	—	14.8% (18)	43.4% (53)	44.8% (50)	100% (122)
Anxiety	—	—	2.3% (1)	68.2% (30)	29.5% (13)	100% (44)
Phobic anxiety	—	—	—	95.7% (22)	4.3% (1)	100% (23)

Total	1.3% (3)	.4% (1)	9.6% (22)	52.0% (119)	36.7% (84)	100% (229)

—: no observation, that is, 0 (.0%).
